# TGF‐β1 rs1982073 polymorphism contributes to radiation pneumonitis in lung cancer patients: a meta‐analysis

**DOI:** 10.1111/jcmm.12933

**Published:** 2016-07-29

**Authors:** Ze‐Tian Shen, Jun‐Shu Shen, Xiao‐Qin Ji, Bing Li, Xi‐Xu Zhu

**Affiliations:** ^1^ Department of Radiation OncologyJinling HospitalMedical School of Nanjing UniversityNanjingChina

**Keywords:** TGF‐β1, polymorphism, lung cancer, radiation pneumonitis, meta‐analysis

## Abstract

Transforming growth factor beta 1(*TGF*‐*β1*) polymorphism was associated with radiation pneumonitis (RP) susceptibility, but their results have been inconsistent. The PubMed and CNKI were searched for case‐control studies published up to Januray 01, 2016 was Data were extracted and pooled odds ratios (OR) with 95% confidence intervals (CI) were calculated. In this meta‐analysis, we assessed eight publications involving 368 radiation pneumonitis cases and 855 controls of the association between *TGF‐β1* T869C (rs1982073) and G915C (rs1800471) polymorphism and RP susceptibility. Our analysis suggested that *TGF‐β1* T869C rs1982073 polymorphism was associated with lower RP risk for CT combined CC *versus* TT model (OR = 0.58, 95% CI = 0.43–0.77). However, for the G915C rs1800471 polymorphism, no association was found between the polymorphism and the susceptibility to RP in GC combined CC *versus* GG model (OR = 0.82, 95% CI = 0.50–1.35). These results from the meta‐analysis suggest that T869C rs1982073 polymorphism of *TGF‐β1* may be associated with RP risk, and there may be no association between G915C polymorphism and RP risk.

## Introduction

Lung cancer remains the deadliest cancer worldwide despite improvements in diagnostic and therapeutic techniques 1. Its incidence has got to peak in many parts of world, particularly in China, which has become a major public health challenge 2. Radiotherapy is an important and commonly used modality in lung cancer treatment, but normal tissues in the vicinity of the target area of the radiation beam are inevitably irradiated 3.

Radiation pneumonitis (RP) is a dose‐limiting factor for radiotherapy and have a major influence on patients prognosis and life quality, which occurs in 5–15% of people who go through radiation therapy for lung cancer. Although many factors can influence the severity of reaction to radiotherapy, a large part of interpatient variability attribute to the individual differences in radio‐sensitivity, which was assumed to be determined by genetic variations among patients 4.

Transforming growth factor beta 1 (*TGF‐β1*) signalling pathway is involved in many cellular processes in both the adult organism and the developing embryo including cell growth, cell differentiation, apoptosis, cellular homeostasis and other cellular functions. *TGF‐β1* gene is one of the most extensively studied cytokines in the development of tissue fibrosis in response to irradiation and *TGF‐β1* signalling is an important modulator of the inflammatory response 5. Previous studies have demonstrated that *TGF‐β1* is a major regulator of radiation‐induced lung injury 6, 7, 8. Radiogenomics with genotyping analysis of SNPs in TGFb1 genes may allow the identification of genotypes prone to RP. Some studies have investigated the associations between the TGF‐β1 polymorphisms and susceptibility of RP 9, 10, 11, 12, 13, 14, 15, 16. However, the results were quite controversial and inconsistent. A single study may be too underpowered to detect a possible small effect of the polymorphisms on lung cancer, especially when the sample size is relatively small. Different types of study populations may also contribute the disparate findings. Hence, we performed a meta‐analysis of all eligible studies to derive a more precise estimation of the correlation between TGF‐β1 polymorphisms and RP risk.

## Materials and methods

### Publication search

Two investigators independently searched the PubMed, Embase and Web of Science databases using the terms ‘polymorphism’ or ‘TGF‐beta’ or ‘*TGF‐β1*’ ‘rs1982073’ or ‘rs1800471’ or ‘Radiation Pneumonia’ or ‘Radiation Pneumonitis’ or ‘toxicity’ or ‘adverse effect’. An upper date limit of Januray 01, 2016 was applied; no lower date limit was used. The search was performed without any restrictions on language and was focused on studies that had been conducted in humans. Concurrently, the reference lists of reviews and retrieved articles were searched manually. Only full‐text articles were included. When the same patient population appeared in several publications, only the most recent or complete study was included in this meta‐analysis.

### Inclusion criteria

For inclusion in this meta‐analysis, the identified articles had to provide information on the following: (1) evaluating the *TGF‐β1* T869C (rs1982073) or G915C (rs1800471) polymorphism and RP risk, (2) case–control studies, and (3) supply the number of individual genotypes for *TGF‐β1* T869C (rs1982073) or G915C (rs1800471) in RP cases and controls, respectively. Major reasons for the exclusion of studies were as follows:(i) duplicate data, (ii) abstract, comment, review and editorial and (iii) no sufficient data were reported.

### Data extraction

Information was carefully extracted from all eligible publications independently by two authors according to the inclusion criteria listed above. The following data were collected from each study: first author's surname, year of publication, ethnicity, total numbers of cases and controls, and numbers of cases and controls with the wild‐type genotypes, the genotypes of homozygote and allele carriers, respectively. If data from any of the above categories were not reported in the primary study, items were treated as ‘not applicable’. Different ethnicity descents were categorized as Asian, and Caucasian. For studies with inadequate information, authors of those studies were contacted for further information by E‐mail if possible.

### Statistical analysis

OR (odds ratio) with 95% CI was used to assess the strength of association between the *TGF‐β1* T869C (rs1982073) or G915C (rs1800471) polymorphisms and RP risk. The pooled ORs for the risk associated with the genotypes of homozygote and allele carriers with the wild‐type genotypes were calculated. Heterogeneity assumption was checked by the chi‐square‐based *Q*‐test 17. A *P* value greater than 0.10 for the *Q*‐test indicates a lack of heterogeneity among studies, so the pooled OR estimate of the each study was calculated by the fixed‐effects model (the Mantel–Haenszel method) 18. Otherwise, the random‐effects model (the DerSimonian and Laird method) was used 19. One‐way sensitivity analyses were performed to assess the stability of the results, namely, a single study in the meta‐analysis was deleted each time to reflect the influence of the individual data‐set to the pooled OR 20. An estimate of potential publication bias was carried out by the funnel plot, in which the standard error of log (OR) of each study was plotted against its log (OR). An asymmetric plot suggests a possible publication bias. Funnel plot asymmetry was assessed by the method of Egger's linear regression test, a linear regression approach to measure the funnel plot asymmetry on the natural logarithm scale of the OR. The significance of the intercept was determined by the *t*‐test suggested by Egger (*P* < 0.05 was considered representative of statistically significant publication bias) 21. All of the calculations were performed using STATA version 11.0 (Stata Corporation, College Station, TX, USA).

## Results

### Study characteristics

A total of eight publications involving 368 radiation pneumonitis cases and 855 controls met the inclusion criteria and were ultimately analysed 9, 10, 11, 12, 13, 14, 15, 16. Tables 1 & 2 present the main characteristics of these studies. Among the six publications, six were published in English and two in Chinese. The sample sizes ranged from 60 to 209. For the population included in the studies, there were three group of China, two groups of USA, one group of Belgium and one group of Arabian.

**Table 1:** Distribution of TGF‐β1 T869C rs1982073 among radiation pneumonitis cases and controls included in this meta‐analysis

First author (year)	Ethnicity (country of origin)	Source of controls	Total sample (case/control)	Cases			Controls		
				TT	TC	CC	TT	TC	CC
Niu (2012)	Asian (China)	Hospital‐based	46/121	8	22	16	20	67	34
Tucker (2012)	Caucasian (USA)	Population‐based	28/113	16	10	2	30	69	14
Voets (2012)	Caucasian (Belgium)	Hospital‐based	59/150	36	16	7	77	59	14
Wang (2010)	Asian (China)	Population‐based	64/115	18	46*		36	79*	
Wang (2011)	Asian (China)	Hospital‐based	38/96	17	12	9	23	44	29
Yuan (2009)	Caucasian (USA)	Hospital‐based	74/89	17	18*		35	93*	
Alsbeih (2010)	Asian (Arabian)	Population‐based	30/30	15	10	5	8	11	11

TT indicates wild‐type, TC indicates heterozygote, CC indicates variant homozygote. *The number of the combined TC and CC genotypes.

**Table 2:** Distribution of TGF‐β1 G915C rs1800471 among radiation pneumonitis cases and controls included in this meta‐analysis

First author‐year	Ethnicity (country of origin)	Total sample (case/control)	Cases			Controls		
			GG	GC	CC	GG	GC	CC
Niu (2012)	Asian (China)	46/121	46	0	0	120	1	0
Tucker (2012)	Caucasian (USA)	28/113	26	2	0	98	12	3
Voets (2012)	Caucasian (Belgium)	59/150	49	10	0	137	13	0
Wang (2011)	Asian (China)	38/96	38	0*		96	0*	
Yuan (2009)	Caucasian (USA)	74/89	33	3*		111	17*	
Zhang (2008)	Asian (China)	29/141	14	7	8	46	72	24

GG indicates wild‐type, GC indicates heterozygote, CC indicates variant homozygote. *The number of the combined GC and CC genotypes.

### Meta‐analysis results

**Figure 1:**
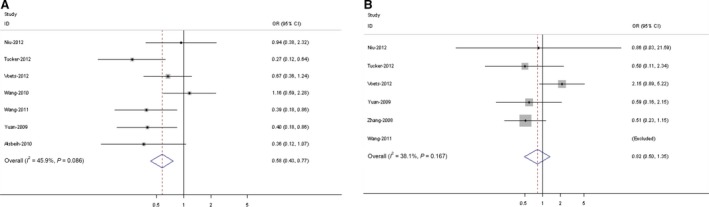
Forest plot (random‐effects model) of radiation pneumonitis risk associated with TGF‐β1 T869C rs1982073 polymorphism for (TC + CC) *versus* TT(**A**). Forest plot (random‐effects model) of radiation pneumonitis risk associated with TGF‐β1 G915C rs1800471 polymorphism for (GC + CC) *versus* GG(**B**). The diamond (and broken line) represents the overall summary estimate, with CI represented by its width. The unbroken vertical line is set at the null value (OR = 1.0).

Seven case**–**control studies with 339 cases and 714 controls were included for association between TGF‐b1 T869C (rs1982073) polymorphism and RP risk. The evaluations of the association of TGF‐b1 T869C polymorphism with RP risk are shown in Figure 1A. The results of the combined analyses showed that TGF‐b1 T869C (rs1982073) polymorphism was associated with lower RP risk for CT combined CC *versus* TT model (OR = 0.58, 95% CI = 0.43–0.77). However, for the G915C (rs1800471) polymorphism, 7 studies with 274 cases and 710 controls were included eventually. No association was found between the polymorphism and the susceptibility to RP in GC combined CC *versus* GG model (Figure 1B, OR = 0.82, 95% CI = 0.50–1.35).

### Publication bias

**Figure 2:**
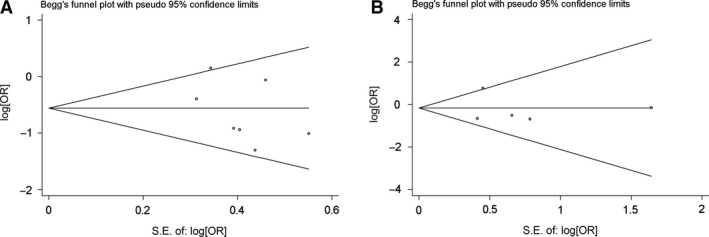
Begg's funnel plot of TGF‐β1 T869C rs1982073 polymorphism and radiation pneumonitis risk for (TC + CC) *versus* TT(**A**). Begg's funnel plot of TGF‐β1 G915C rs1800471polymorphism and radiation pneumonitis risk for (GC + CC) *versus* GG(**B**).

Begg's funnel plot and Egger's test were performed to access the publication bias of literatures. Evaluation of publication bias for the T869C (rs1982073) polymorphism showed that the Egger test was not significant (*P* = 0.421). The funnel plots for publication bias (Figure 2A) also did not show some asymmetry. For G915C (rs1800471) polymorphism showed that the Egger test was not significant (*P* = 0.245). The funnel plots for publication bias (Figure 2B) also did not show some asymmetry. These results did not indicate a potential for publication bias.

## Discussion

RP is an inflammation of the lungs due to radiation therapy. Because current parameters for predicting RP are very limited, there is a demand for the possibility of developing novel parameters. Although many factors can influence the severity of reaction to radiotherapy, a large part of interpatient variability attribute to the individual differences in radiosensitivity, which was assumed to be determined by genetic variations among patients. Recently, studies have focused on the association of common polymorphic variations in candidate genes and individual differences in radiosensitivity 22.

The T869C (rs1982073) and G915C (rs1800471) polymorphisms of *TGF‐β1* lead to amino acid substitutions in *TGF‐β1*, which may alter *TGF‐β1* function. This change in protein biochemistry leads to the supposition that variant alleles may diminish repair kinetics, thereby influencing susceptibility to adverse health effects 23. Some researchers reported that the return of plasma *TGF‐β1* levels to normal after radiotherapy accurately predicted that patients would not develop RP 24. In recent years, several molecular epidemiological studies have been conducted to evaluate the role of polymorphisms in the *TGF‐β1* on RP susceptibility in lung cancer patients; however, the results remain conflicting rather than conclusive.

In recent years, several molecular epidemiological studies have been conducted to evaluate the role of polymorphisms in the *TGF‐β1* on RP susceptibility in lung cancer patients; however, the results remain conflicting rather than conclusive. Wang *et al*. 25 had performed a meta‐analysis about similar subjects based on published data updated in August 2014, which concluded that *TGF‐*869C/T polymorphism was a risk factor of RP. Though that results were consistent with ours, only five studies were included in the meta‐analysis, while our study included eight studies and focused two genotypes of *TGF‐β1*. We have improved upon that previous meta‐analysis by including more recent related studies and by generally using a more comprehensive search strategy. We also explored heterogeneity and potential publication bias in accordance with published guidelines. In this meta‐analysis, we found that *TGF‐β1* T869C (rs1982073) polymorphism may be associated with RP susceptibility, and there may be no association between G915C (rs1800471) polymorphism and RP susceptibility.

The results of combined analyses suggested that the *TGF‐β1*T869C (rs1982073) polymorphism was associated with an increased RP susceptibility, while the *TGF‐β1* G915C (rs1800471) was not associated with RP susceptibility when all the studies were pooled. These results implied that genetic factors may have played a greater role in influencing individual patient susceptibility, suggesting the possibility of using these biomarkers as predictive factors. In additional, future understanding of the combined effect of these polymorphisms on patient response to radiotherapy may shed some light on the predictive value of these genetic factors.

Some limitations of this meta‐analysis should be acknowledged. Firstly, we did not perform subgroup analysis by the pathological types of lung cancer due to limited data in primary studies. Because of different pathological types, subgroup analysis should be performed. However, some study in this meta‐analysis did not report separate genotype frequency for each pathological type of lung cancer, which prevented us to perform this subgroup analysis. Secondly, only published studies were included in this meta‐analysis. The presence of publication bias indicates that non‐significant or negative findings may be unpublished. Thirdly, the radiotherapy parameters, including total dose, dose per fraction, field size, irradiation volume and depth of prescription point, were not identical. The majority of the treatment protocols in the included studies were based on multimodality treatment that is an important potential confounding factor aggravating the adverse effects, particularly when adjuvant or concurrent chemotherapy was involved. Lastly, our results were based on unadjusted estimates, while a more precise analysis should be conducted if individual data were available, which would allow for the adjustment by other covariates including age, family history, environmental factors and lifestyle.

In conclusion, this meta‐analysis suggests that *TGF‐β1* T869C (rs1982073) polymorphism may be associated with RP risk. In addition, it is necessary to conduct large trials using standardized unbiased methods, homogeneous lung cancer patients and well‐matched controls, with the assessors blinded to the data. These patients might benefit from individual radiotherapy regimens and early intervention for adverse effects accordingly.

## Conflict of interest

The authors confirm that there are no conflicts of interest.
